# Characteristics, pathogenic mechanism, zoonotic potential, drug resistance, and prevention of avian pathogenic *Escherichia coli* (APEC)

**DOI:** 10.3389/fmicb.2022.1049391

**Published:** 2022-12-13

**Authors:** Jiangang Hu, Dossêh Jean Apôtre Afayibo, Beibei Zhang, Hong Zhu, Lan Yao, Weiqi Guo, Xinyu Wang, Zhiyang Wang, Di Wang, Haoheng Peng, Mingxing Tian, Jingjing Qi, Shaohui Wang

**Affiliations:** Shanghai Veterinary Research Institute, Chinese Academy of Agricultural Sciences (CAAS), Shanghai, China

**Keywords:** APEC, epidemiology, virulence, zoonotic, antibiotic resistance, control strategies

## Abstract

Although most *Escherichia coli* (*E. coli*) strains are commensal and abundant, certain pathogenic strains cause severe diseases from gastroenteritis to extraintestinal infections. Extraintestinal pathogenic *E*. *coli* (ExPEC) contains newborn meningitis *E*. *coli* (NMEC), uropathogenic *E*. *coli* (UPEC), avian pathogenic *E*. *coli* (APEC), and septicemic *E*. *coli* (SEPEC) based on their original host and clinical symptom. APEC is a heterogeneous group derived from human ExPEC. APEC causes severe respiratory and systemic diseases in a variety of avians, threatening the poultry industries, food security, and avian welfare worldwide. APEC has many serotypes, and it is a widespread pathogenic bacterium in poultry. In addition, ExPEC strains share significant genetic similarities and similar pathogenic mechanisms, indicating that APEC potentially serves as a reservoir of virulence and resistance genes for human ExPEC, and the virulence and resistance genes can be transferred to humans through food animals. Due to economic losses, drug resistance, and zoonotic potential, APEC has attracted heightened awareness. Various virulence factors and resistance genes involved in APEC pathogenesis and drug resistance have been identified. Here, we review the characteristics, epidemiology, pathogenic mechanism zoonotic potential, and drug resistance of APEC, and summarize the current status of diagnosis, alternative control measures, and vaccine development, which may help to have a better understanding of the pathogenesis and resistance of APEC, thereby reducing economic losses and preventing the spread of multidrug-resistant APEC to humans.

## Introduction

*Escherichia coli* (*E. coli*) colonizes the gastrointestinal tract and other mucosal surfaces of a variety of animals (Hill and Drasar, 1975). Although most *E. coli* strains are commensal and abundant, certain pathogenic strains can cause severe diseases from gastroenteritis to extraintestinal infections that affect health worldwide (Russo and Johnson, 2003). According to the anamnestic clinical reports and virulence features, pathogenic *E. coli* have been classified as either intestinal pathogenic *E. coli* (IPEC) or extraintestinal pathogenic *E. coli* (ExPEC) (Russo and Johnson, 2003). Six diarrhoeagenic pathovars of IPEC have been extensively studied. ExPEC contains newborn meningitis *E. coli* (NMEC), uropathogenic *E. coli* (UPEC), avian pathogenic *E. coli* (APEC), or septicemic *E. coli* (SEPEC) based on their original host and clinical symptoms. Among these typical infections caused by ExPEC in humans are urinary tract infections (UTIs) and neonatal meningitis (Russo and Johnson, 2000). Similarly, APEC is mostly associated with respiratory tract or systemic infections and results in a variety of diseases in chickens, ducks, and other avian species worldwide, which are economically devastating to poultry industries. There is an increasing risk of ExPEC due to its abundance and multidrug resistance (Antão et al., 2008).

More and more shreds of evidence indicated that ExPEC strains involved in animal and human infections have a highly similar range of phylogenetic and pathogenic mechanisms (Moulin-Schouleur et al., 2007; Reid et al., 2019). Moreover, the whole-genome sequencing of *E. coli* strains indicated that the genomic level of human ExPEC strains is clustered with avian isolates. Some *E. coli* strains could acquire a combination of mobile genetic elements via a horizontal exchange, to become a highly adapted pathogen capable of survival and causing a range of diseases in humans and animals (Shames et al., 2009). Thus, the APEC might be virulence genes and antibiotic-resistant genes reservoir for human ExPEC strains. It is necessary to consider the zoonotic potential of APEC (Manges and Johnson, 2012). Although the pathogenic mechanisms of APEC have not yet been completely elucidated, insights into virulence factors of APEC are increasing, which helps to develop novel strategies for controlling APEC infections. In this review, we highlight the recent advances in zoonoses’ potential characteristics, antibiotic resistance, and control strategies of APEC to provide guidance for the prevention and control of avian colibacillosis.

## Avian colibacillosis associated with avian pathogenic *Escherichia coli*

Avian colibacillosis is an assembly of many extraintestinal infections in chickens and other birds with APEC as etiological agents (Barbieri et al., 2015). APEC is susceptible to inducing localized and systemic types of colibacillosis with two important infection stages. The primary stage of colibacillosis was identified as infections of the reproductive tract, omphalitis, and yolk sac. Salpingitis-peritonitis-salpingoperitonitis syndrome (SPS) causes reproductive tract infections with multiple and specific symptoms (Johnson et al., 2008). Omphalitis and yolk sac infection, which are caused by fecal contamination of eggs or egg formation, affect chicks with high mortality in poultry (Matthijs et al., 2009). APEC, in the secondary colibacillosis infections stage, assures an important role in bone and joint infections affecting poultry flocks (Nolan et al., 2003). Among the several types of colibacillosis, colisepticemia was identified as the most important systemic form. Colisepticemia occurs in birds under stress and weak immunosystems through the degradation of certain biotic and abiotic factors mostly high humidity, excess temperature, high dust, viral infections, and vaccines or virulent infectious bronchitis virus in the poultry (Matthijs et al., 2009).

## Epidemiology of avian pathogenic *Escherichia coli*

Avian pathogenic *E*. *coli* has been known and reported as a principal etiologic agent of avian colibacillosis, responsible for significant morbidity and mortality, with resultant serious economic losses to the poultry industry in the world (Ronco et al., 2017). According to its impact worldwide, serotyping was the most approved method used frequently to estimate the pathogenic potential of APEC strains. *E. coli* strains belonging to somatic (O), capsular (K), and flagellar (H) antigens. The specific O-serotypes have close correlations with pathogenic *E. coli* strains (Kauffmann, 1947). Previous studies indicated that O78, O1, and O2 were the predominant serotypes of APEC, whereas, there are different prevalent serotypes in diverse countries according to geographic distribution (Ewers et al., 2004). A recent report that O145 may be emerging as a predominant serogroup of APEC in China (Wang et al., 2022). In addition, the *E. coli* strain could be assigned to one of the main phylo-groups (A, B1, B2, and D) (Clermont et al., 2000). Significantly, strains responsible for extraintestinal infection were far more likely to be members of phylo-groups B2 or D than A or B1 (Johnson and Stell, 2000). The ExPEC, including APEC strains, mainly belong to the phylogroup B2 and a lesser extent to group D (Smith et al., 2007).

## Pathogenic mechanism and virulence factors of avian pathogenic *Escherichia coli*

### Pathogenic mechanism

**FIGURE 1:**
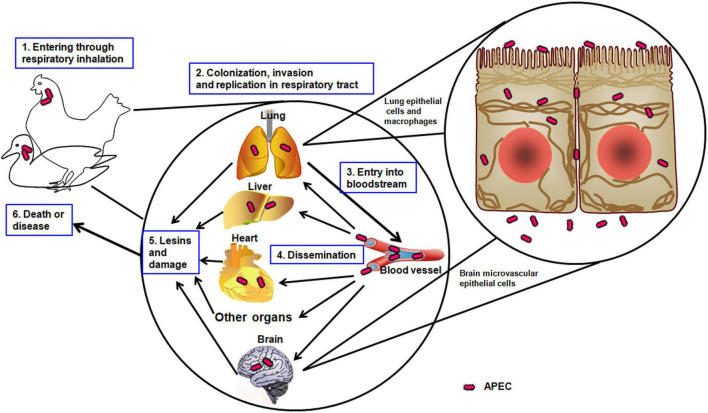
The pathogenic mechanism of APEC. The schematic illustrates the APEC infection progress in avians, such as chickens or ducks.

Colibacillosis is an important part of a respiratory infection that evolves to generalize infection resulting in fibrinopurulent lesions of internal organs (Kathayat et al., 2021). Pathogenic bacteria use many strategies to sustain themselves and overcome host barriers with the adhesion of the microorganism to host cells (Mellata, 2013). Colonization is a common step in the pathogenesis of pathogenic bacteria through the ability to adhere to the host surfaces and the successful replication in the respiratory tract (Mellata, 2013). APEC enters through the respiratory tract and uses adhesins to attach to the epithelial cells, followed by survival, invasion, and replication via the presence of the invasins and complementary defense mechanisms (Figure 1). Then, APEC enters the bloodstream, disseminates through the vital organs, such as the lung, heart, liver, and brain, and causes significant damage and lesions (Pourbakhsh et al., 1997). Finally, APEC leads the host to death or induces illness. The resistance to phagocytosis may be an important mechanism in the development of colisepticemia when a strong correlation was observed between pathogenicity for chickens *in vivo* and the ability to resist the bactericidal effects of chicken macrophages *in vitro* (Dho-Moulin and Morris Fairbrother, 1999).

### Virulence factors of avian pathogenic *Escherichia coli*

During the process of APEC infection, various virulence factors, including adhesins, iron acquisition systems, protectins, toxins, invasins, metabolism, and secretion systems (Table 1), play important roles in the colonization and survival of APEC (Li et al., 2010).

**TABLE 1:** Validated virulence factors in APEC.

Name/Description	Functions	Present in ExPEC	References
**Adhesins**			
Type I fimbriae	Colonization, biofilm formation	APEC, NMEC, SEPEC UPEC,	Ewers et al., 2007
P fimbriae	Colonization, stimulate of cytokines production,	APEC, UPEC, SEPEC,	Kariyawasam and Nolan, 2009
Flagella (FliC)	Colonization, biofilm formation	APEC, UPEC	Dziva et al., 2013
Curli	Colonization, biofilm formation	APEC, UPEC, SEPEC	La Ragione et al., 2000
Temperature sensitive hemagglutinin (Tsh)	Adherence	APEC, UPEC, NMEC	Kostakioti and Stathopoulos, 2004
**Iron acquisition**			
Aerobactin	Siderophore, acquisition of iron	APEC, UPEC	Gao et al., 2015a
Salmochelin	Siderophore receptor, use of Fe irons	APEC, NMEC, SEPEC, UPEC	Caza et al., 2008
SitABC	Transportation of Fe, Mn	APEC, UPEC	Sabri et al., 2008
**Antiphagocytic activity/serum resistance**			
Transfer protein	Inhibition of the classical pathway of complement activity	APEC, NMEC, SEPEC	Sarowska et al., 2019
Capsule	Resistance to human alpha-defensin 5	APEC, NMEC, UPEC	Thomassin et al., 2013
LPS	Reduce the environmental acidity	APEC	Yu et al., 2015
Increased serum survival (Iss)	Protect against phagocytosis	APEC, NMEC, SEPEC	Nolan et al., 2003
**Toxins**			
Vacuolating autotransporter toxin	Induce vacuolization of host cell	APEC, UPEC	Ewers et al., 2004
Serin protease autotransporter	Epithelium colonization, mucins degradation	APEC, UPEC	Pokharel et al., 2019
**Invasins**			
IbeA	Invasion, resistance to oxidative stress	APEC, NMEC, SEPEC	Cortes et al., 2008
IbeB	Invasion	APEC, NMEC	Wang et al., 2012
GimB	Adherence and invasion	APEC, NMEC, UPEC	Matter et al., 2015
**Two component regulatory systems**			
CpxA/CpxR	Fitness, virulence	APEC, UPEC	Yamamoto and Ishihama, 2006
BarA/UvrY	Biofilm formation, persistence	APEC, UPEC	Herren et al., 2006
RstA/RstB	Acid resistance, intracellular survival	APEC	Gao et al., 2015b
PhoB/PhoR	Intracellular survival	APEC	Bertrand et al., 2010
**Secretion systems**			
*E. coli* type III secretion system 2 (ETT2)	Virulence, intracellular survival	APEC, NMEC, UPEC	Wang et al., 2016a; Fu et al., 2021; Li et al., 2022; Xue et al., 2022; Yin et al., 2022
Type VI secretion system	Interbacterial competition, stress sensing, virulence	APEC, NMEC	Hachani et al., 2016

### Adhesins

Adhesins are a cell-surface system of bacteria that adhere to the epithelial cells during the initial stages of APEC infections (Kalita et al., 2014). Initial bacterial attachment or adhesion to host cells is vital to bacterial pathogenesis and is determined by various adhesins. There are many adhesins in APEC, including type 1 fimbriae, P fimbriae, S fimbriae, flagella, curli, outer membrane protein, non-fimbrial and atypical adhesins, and temperature-sensitive hemagglutinin (Aleksandrowicz et al., 2021). Type 1 fimbriae facilitate the adherence to epithelial cells of the respiratory tract during the primary stage (Ewers et al., 2007), whereas the expression of P fimbriae and S fimbriae contribute to the late infection. The application of normal anti-type 1 fimbriae serum and D-mannose, which are the cellular receptors of the adhesin of type 1 fimbriae, could block the specific adherence of APEC strains to chicken tracheal sections (Gyimah and Panigrahy, 1988). Curli induces resistance of APEC in the host’s cecum and facilitates the bacterial invasion of the whole cells (Gophna et al., 2001). Temperature-sensitive hemagglutinin (Tsh) intervenes in the colonization in the first stage of respiratory tract infection (Kostakioti and Stathopoulos, 2004).

### Iron acquisition systems

Iron acquisitions have a large operating system and contribute to the growth and proliferation of APEC in the host (Rodriguez-Siek et al., 2005b). Multiple siderophores, including aerobactin, salmochelin, yersiniabactin, and transporters to sequester iron from the body fluids, were reported in the APEC. In APEC, the proportion of these operons is increased and located on large plasmids (Johnson et al., 2006). APEC strains have an important frequency of aerobactin iron acquisition system compare to non-pathogenic strains (Dozois et al., 1994).

### Protectins

Protectins are structural factors, including the K1 capsule, lipopolysaccharide (LPS), and certain outer membrane proteins (OMPs), which protect bacteria from the host immune system under stress conditions (Mellata et al., 2003). The capsule is an important virulence factor for the spread of APEC in the bloodstream and septicemia (Biran and Ron, 2018). Furthermore, the *iss* gene present in the ColV plasmid plays an important role in the bacteria serum survival. In addition, protectins also mediate APEC adhesion, invasion, intracellular survival, colonization, and proliferation in the host (Huja et al., 2015).

### Toxins

Toxins are biologically harmful substances that intervene in the bacterial ability to invade and cause damage to the tissues. APEC produces a low quantity of toxins, including vacuolating autotransporter toxin (Vat), cytotoxic necrotizing factor 1 (CNF1), and various hemolysins (Rodriguez-Siek et al., 2005a). These toxins play role in colonization, motility, biofilm formation, agglutination, and induction of vacuolization. Vat causes cytotoxic effects in cultured cells and attenuates virulence when its deleted (Parreira and Gyles, 2003).

### Invasins

Invasins facilitate the entrance of APEC into the host cells during the infection. Several genes encoding invasins, *ibeA*, *ibeB*, and *gimB* have been identified in NMEC, which contributed to the invasion of the brain microvascular endothelial cells (BMECs) (Germon et al., 2005). These invasins were present in APEC and contributed to resistance to oxidative stress, biofilm formation, colonization, and proliferation in the host (Germon et al., 2005; Wang et al., 2012).

### Secretion systems

Secretion systems are needle-like structures used to secrete effector proteins, which contribute to bacterial survival and virulence (Wang et al., 2016a). Among the different bacterial secretion systems, two important secretion systems (types III and VI) were identified in APEC (Ma et al., 2014). Type III secretion system 2 (ETT2) is found frequently in pathogenic *E. coli* strains. The O1, O2, and O78 serotypes of APEC were identified as possessing important elements of ETT2 (Wang et al., 2016a). The intact and degenerate forms are identified in the O1, O2, and O78 serotypes (Wang et al., 2016b). However, the degenerative form of ETT2 may contribute to reducing the virulence and serum survival activity in bacteria (Wang et al., 2016a). Multiple components of ETT2 are involved in the pathogenicity of APEC (Wang et al., 2016a; Fu et al., 2021; Tu et al., 2021; Li et al., 2022; Xue et al., 2022; Yin et al., 2022). Our research shows that the transcriptional regulator DctR can regulate the expression of ETT2 and affect the virulence and pathogenicity of APEC (Zhang et al., 2021). In all, ETT2 has been discovered in APEC isolates and plays significant roles in bacterial virulence, adhesion, colonization, intracellular survival, serum bactericidal activity, and the downregulation of pro-inflammatory cytokine responses. The type VI secretion system (T6SS) is one of the recent nanomachine secretion systems present in Gram-negative pathogens (Yi et al., 2019). Two different forms such as multipurpose T6SS1 and conservative T6SS2 were discovered in APEC isolates. The T6SS1 intervened in the proliferation of APEC during infection, whereas T6SS2 played a role only for cerebral infection (Ma et al., 2014). Overall, the type VI secretion systems are specialized in interbacterial competition, stress sensing, biofilm formation, and virulence (Hachani et al., 2016). In addition, APEC and NMEC have similar T6SS which contributes to binding, and competition by using it to kill neighboring non-immune bacteria and pathogenesis of APEC and NMEC (Ma J. et al., 2018).

### Two-component regulatory systems

Two-component systems (TCSs) are signaling proteins that play important roles in modulating bacterial fitness in different niches. Different TCSs such as CpxA/CpxR, BarA/UvrY, RstA/RstB, and PhoB/PhoR have been identified in APEC isolates (Tu et al., 2016). The CpxA/CpxR regulates surface structure assembly and stress response system implicated in APEC. In addition, CpxA/CpxR positively controls the expression of the APEC type VI secretion system 2 (Yi et al., 2019). BarA/UvrY regulates virulence properties in APEC through the adhesion, invasion, persistence, intracellular survival, resistance to serum bactericidal activity and oxidative stress, and regulation of exopolysaccharide production and expression of type 1 and P fimbriae (Palaniyandi et al., 2012). The RstA/RstB is a nitrogen metabolism TCS that contributes to iron acquisition, acid resistance, intracellular survival, and colonization (Gao et al., 2015b). The PhoB/PhoR is present in many bacterial species that respond to external phosphate concentrations and intervene in biofilm formation, motility, adhesion, invasion, and systemic dissemination (Bertrand et al., 2010).

## The zoonotic potential of avian pathogenic *Escherichia coli* increases the risk of resistance

### Avian pathogenic *Escherichia coli* is a potential reservoir for the contamination of human ExPEC

Several studies have shown the phylogenic similarity between APEC and human ExPEC isolates. According to phylogenetic classification, APEC isolates share significant genetic similarities with human ExPEC (Johnson et al., 2007). ExPEC (APEC, UPEC, and NMEC) share the same virulence factors and similar pathogenic mechanisms, and it may be spread between animals and humans (Johnson et al., 2008). The report demonstrated that ExPEC strains including APEC derived from specific STs may have a high zoonotic impact on humans (Johnson et al., 2008). Several specific virulence genes of the APEC strain, detected in UPEC plasmids, were shown susceptible to increasing the bacteria process to get iron in deficiency conditions (Ewers et al., 2007). Virulence genes that operate in ColV plasmids have similar functions in APEC and UPEC strains (Johnson et al., 2008). Moreover, APEC may induce high urinary infections in mice similar to UPEC and meningitis in rats similar to NMEC (Johnson et al., 2010). In addition, the ExPEC was recognized as a potential causal agent in women’s health, newborns, elderly, and immunocompromised individuals in fact of an important number of urinary tract infections (UTIs), newborn meningitis, abdominal sepsis, and septicemia (Mellata, 2013).

### As a foodborne pathogen, the emergence and transfer of antibiotic-resistance genes

Poultry is one of the most widely consumed meats in the world. APEC are causative agents of colibacillosis, one of the principal causes of morbidity and mortality in poultry worldwide (Nhung et al., 2017). Since poultry is usually raised under intensive conditions, infection transmission is favored, and the animals are more susceptible to diseases. So using large quantities of antimicrobials to prevent and treat disease, if overused or misused, lead to the evolution of bacteria and the rise of drug-resistant pathogens in the long term (Rahman et al., 2022). It is precisely due to the extensive use of antibiotics that APEC is severely resistant. Antibiotic-resistant APEC can not only lead to treatment failure, resulting in economic losses, but also be a source of resistant bacteria/genes that may represent a risk to human health (Nhung et al., 2017; Kim et al., 2021).

Avian pathogenic *E*. *coli* already carries many resistance genes and resistance to a lot of important antibiotics around the world. Colistin resistance of 2.2% was detected in isolates in Senegal, and colistin resistance of 8.7% was detected in isolates in Vietnam (Vounba et al., 2019). Particularly, there was a higher prevalence of *mcr-1* in isolates from chicken in Vietnam (53.2%), and the *mcr-1* gene was detected in 85% of 13 phenotypically colistin-resistant isolates (Vounba et al., 2019; Le et al., 2021). In addition, all colistin-resistant isolates exhibited multidrug-resistant phenotypes (Vounba et al., 2019). In Jordan, APEC resistance rates of sulfamethoxazole–trimethoprim, florfenicol, amoxicillin, doxycycline, and spectinomycin were 95.5, 93.7 93.3, 92.2, and 92.2%, respectively. At least five antibiotic-resistance genes were found in 68% of APEC isolates. The most important genes were *int1* 97%, *tetA* 78.4%, *bla*TEM 72.9%, *Sul1* 72.4%, and *Sul2* 70.2%; these resistance genes are detected in human pathogens (Ibrahim et al., 2019). Under commercial conditions in Portugal, the overall 10-year antibiotic resistance of APEC strains is amoxicillin 78%, ampicillin 73.5%, tetracycline 63.3%, doxycycline 56.4%, apramycin 34.5%, neomycin 68.2%, flumequine 39.4%, cotrimoxazole 47.7%, florfenicol 46.6%, and lincospectin 66.3% (Oliveira et al., 2022). In China, the prevalence of extended-spectrum cephalosporin-resistant strains in *E. coli* from chicken colibacillosis and raw meat separately accounted for 66.1% and 71.2% (Wang et al., 2021).

Previous studies indicated that APEC strains found in poultry are shown to be important reservoirs for antibiotic resistance genes (Nandi et al., 2004). Antibiotic resistance occurs with high complexity in presence of resistance-encoding genes that are found inside plasmids or chromosomal genetic material (Ibrahim et al., 2019). The antibiotic resistance genes are identified on mobile genetics elements enabling their rapid transfer among the ExPEC strains. Furthermore, animal reservoirs may be responsible for human contamination or transfer of APEC antibiotic-resistant and other commensal bacteria through the contaminated food in poultry (Hannah et al., 2009). Investigations from the poultry farm revealed the use of multiple antibiotics may present significant resistance among *E. coli* (Johar et al., 2021). The *mcr-1* gene, found in APEC, exhibited colistin resistance, demonstrating its role in colistin resistance (Eltai et al., 2018). In addition, APEC carrying the *mcr-1* gene was isolated from septicemic chickens, which may increase the difficulty of prevention and control of poultry septicemia (Ewers et al., 2016). The research has shown that β-lactamase *CTX-M*, *OXA*, *CMY*, and *TEM* genes were widespread in chicken-source *E. coli*, and *bla*CTX-M was the most predominant ESBL gene (Wang et al., 2021). The studies identify resistance genes such as *floR, cmlA, cat1, cat2, tetA, tetB, tetC, tetD, tetE, tetG, sul1, sul2, addA1*, and *addA2* among APEC isolates (Li et al., 2007; Shin et al., 2015; Ibrahim et al., 2019). The presence of the resistance gene in isolates from poultry and marketed retail meats further complicates the APEC antibiotic resistance situation and is a possible health risk for humans.

## Diagnosis, prevention, and control of avian pathogenic *Escherichia coli* infection

### Diagnosis

Colibacillosis infections are suspected to focus on the clinical signs and the presence of typical macroscopic lesions (Gomis et al., 1997). The laboratory diagnosis must be confirmed in the presence of such clinical signs and lesions, including traditional bacterial isolation, virulence gene detection, and serotyping. The diagnosis occurs in different regions of an infected animal such as cardiac blood and affected tissues, liver, spleen, and pericardium. Samples of the lesion were collected and prepared. Selective media like McConkey, eosinmethylene blue, or drigalki agar are used for isolation. The antigenic identification and virulence genes of isolated strains were detected by PCR; specific antigen and virulence gene detection is beneficial to identify APEC (Antão et al., 2009). Traditional biochemical reactions and ELISA methods for APEC identification are costly and time-consuming. Therefore, the PCR method has been found to be a fast and effective common technique for APEC detection (Wang et al., 2014; Lucas et al., 2022).

### Prevention and control

While some families of antibiotics used as a treatment in poultry such as tetracyclines, penicillins, and aminoglycosides are also commonly administered to humans to treat bacterial infections. Therefore, the drug resistance of APEC will threaten the choice of drugs when human beings are infected with bacteria. Other multitude methods such as biosecurity measures and vaccination are also necessary for the prevention and control of infections (Wang et al., 2017).

### Management and biosecurity measures

Effective prevention and control of APEC infections depend on the identification and elimination of predisposing causes of the disease. Maintaining flock biosecurity is difficult to control and prevent (Dziva and Stevens, 2008). The main objective is to reduce the level of APEC exposure by improving biosecurity, good litter, and ventilation conditions in poultry (Dziva and Stevens, 2008). The sanitation of the environmental system should be improved. Furthermore, the reducing of fecal contamination of eggs, cleaning nest boxes, and decreasing the number of floor eggs contribute to reducing the incidence risk of colibacillosis infections (Dziva and Stevens, 2008). The research shows that vitamin E has been able to interfere with bacterial biofilm and prevent *in vitro* biofilm formation (Vergalito et al., 2018). It is possible to increase the level of vitamin E in the nutrition system to prevent APEC infection.

### Antibiotics for treatment

Generally, antibiotics are wildly used to prevent and treat APEC infections. The application of these antibiotics was reported to accelerate the emergence of multidrug-resistant bacteria (Rahman et al., 2022). Seriously, APEC’s high levels of resistance to important antibiotics may pose a high risk to humans, because antibiotic-resistant bacteria and genes can be transmitted through the food chain to humans. Previous studies demonstrated that APEC isolates were resistant to multiple antibiotics. Thus, it is crucial and helpful to perform antibiotic susceptibility testing for the appropriate antibiotic in the treatment of avian colibacillosis (Bass et al., 1999).

### Vaccines

Avian pathogenic *E*. *coli* infections of poultry result in significant morbidity and mortality with important economic losses. Therefore, many efforts have been made to develop effective vaccines, including inactivated vaccines, subunit vaccines, and live attenuated vaccines against APEC infections (Nesta and Pizza, 2018). Table 2 shows the of vaccines development against APEC infection with their main findings.

**TABLE 2:** Summary of vaccines development against APEC infection.

Antigens^a^	Immunity route^b^	Challenge route^b^	Outcome by homologous challenge^c^	Outcome by heterologous challenge^d^	References
**Inactivated vaccine**					
O78	SC, IM, IP	IM or IV	Protective	Not protective	Deb and Harry, 1976
O2	SC, IM	SC	Protective	Not protective	Deb and Harry, 1978
O1	SC	Air sac	Protective	Not protective	Gyimah and Panigrahy, 1985
O1, O2, and O78	IM	N/A	Protective	N/T	Panigraphy et al., 1984
O2; O78	SC, SC	SC; SC	Protective	Protective; not protective	Melamed et al., 1991
O2, O78, and O35	SC	IT	Protective	Not protective	Rosenberger et al., 1985
**Subunit vaccines**					
Aerobactin	IM	Aerosol	Protective	Not protective	Le Roy et al., 1995
IROMPs	IV	Air sac	Protective	N/T	Bolin and Jensen, 1987
SRP	SC	IV	Protective	Protective	Russo et al., 2003
Pilus	SC	Air sac	Protective	N/T	Gyimah and Panigrahy, 1985
FimA	IM	Air sac	Protective	Protective	van den Bosch et al., 1993
FimH	IM	Air sac or aerosol	Not protective	Not protective	Kariyawasam et al., 2002
PapG	IM	Air sac	Protective	Protective	Kariyawasam et al., 2002
IutA	IM	Air sac	Protective	Protective	Kariyawasam et al., 2002
Iss	IM	Air sac	Protective	Protective	Lynne et al., 2012
rOmpA and rFliC, recombinant GroEL	IM	Sterile water	Protective	Not protective	Bao et al., 2013
**Live attenuated vaccines**					
Non-pathogenic *E. coli* O78	Aerosol	Aerosol	Not protective	N/T	Azeem et al., 2017
Non-pathogenic piliated *E. coli* (BT-7)	Aerosol, drinking water	IT	N/A	Protective	Ghunaim et al., 2014
ΔcarAB	IM	IM	N/A	Protective	Frommer et al., 1994
Δcya Δcrp	Oral	IT	Protective	N/T	Kwaga et al., 1994
Δcya Δcrp	Spray, Oral	IT	Protective	N/T	Roland et al., 1999
ΔgalE	Spray	Aerosol	Protective	N/T	Peighambari et al., 2002
DE17ΔaroAΔluxS	Spray	Aerosol	Protective	Not protective	Kariyawasam et al., 2004
ΔpurA	IM	Aerosol	Protective	Protective	Holden et al., 2014
ΔaroA	Spray	Aerosol	Not protective	Not protective	Kariyawasam et al., 2004
O78:K80	Spray	Aerosol	Protective	Not protective	Kariyawasam et al., 2004
**Other vaccines**					
Bacterial ghost	Spray, IM	Air sacs, IM	Protective	Not protective	Ebrahimi-Nik et al., 2018; Hu et al., 2019
*Salmonella* delivery FimA, OmpC, O78	Oral gavage	IM or air sac	Protective	Protective	Han et al., 2018
*Lactobacillus* strains expressing PapA, PapG, IutA, CS31A	Intragastric	Oral	Protective	Not protective	Ma S. T. et al., 2018
Outer membrane vesicles	IM	Air sac	Protective	Not protective	Wang et al., 2019

^a^SRP, siderophore receptor protein; IROMPs, iron-regulated outer membrane proteins; Iss, increased serum survival protein.

^b^IV, intravenous; IM, intramuscular; SC, subcutaneous; IP, intraperitoneal; IT, intratracheal; N/A, not applicable or not available.

^c^N/A, not applicable or not available.

^d^N/T, not tested; N/A, not applicable or not available.

### Inactivated vaccines

Inactivated vaccines were developed earlier to provide the effectiveness of vaccines against homologous and heterologous challenges (Gross, 1957). These vaccines were made from inactivated predominant APEC serotype strains to control colibacillosis. The inactivated vaccines were observed to provide efficacy protection against only homologous challenges (Deb and Harry, 1978). The efficacy of the inactivated vaccines is determined by diverse parameters such as the serotypes of *E. coli* include in the vaccine, the administration methods, age of the birds, and the dose of vaccine administered to the birds (Russo et al., 2003).

### Subunit vaccines

Subunit vaccines were produced to overcome the limit of inactivated vaccines, which were unable to protect chickens against the heterologous challenge (Bolin and Jensen, 1987). Several recombinant subunit antigens intervene to produce subunit vaccines for successful protection against heterologous challenges (Bolin and Jensen, 1987). The recombinant subunit vaccines generate strong antibody responses in recipient birds when administered parenterally with adjuvant-containing formulations (Kariyawasam et al., 2002). Subunit vaccines may provide broader protection to more serotypes of APEC. The understanding of APEC genome sequences and pathogenic virulence genes may contribute to the development of more new subunit vaccines (Ghunaim et al., 2014).

### Live-attenuated vaccines

Live vaccines are available for numerous viral, bacterial, and coccidial organisms. Live vaccines are effective and relatively economical (Frommer et al., 1994). The most successful APEC live attenuated vaccine is the *aroA* gene mutant vaccine. The live Poulvac^®^ *E. coli* (Zoetis) vaccine includes an *aroA* mutant of a strain of serotype O78:K80 and ST23, and the *aroA* mutation attenuates the virulence of the strain and results in a requirement for aromatic amino acids, which results in reduced survival of the strain in the chicken and the environment (La Ragione et al., 2013; Han et al., 2015). The live vaccines are generally short-lived after first or initial exposure to the immunity system. These vaccines generally reduced the systemic lesions by mass administered drinking water, spray, and oral (Barbieri et al., 2012). Even though the live attenuated vaccine provides clinical protection against the challenge, it is not able to prevent completely pathological lesions (El-Mawgoud et al., 2020). Live vaccines may induce a high risk of reversion to natural virulence via back-mutations of the attenuated organism and susceptible to causing symptomatic affection (Ghunaim et al., 2014).

#### Other vaccines

After evaluating the efficacy of inactivated vaccines, subunit vaccines, and live attenuated vaccines, various recombinant vaccines have been investigated to protect chickens against APEC infections (Ghunaim et al., 2014). Within the tested vaccines, multiple vaccines such as outer membrane vesicles (OMVs), bacterial ghost (BG) vaccines, *Salmonella*-delivered vaccines containing APEC antigens, such as FimA and OmpC, were able to reduce the mortality and morbidity, APEC lesions as well as stimulate the antibody (immunoglobulins; IgG and IgA) responses in immunized chickens (Ebrahimi-Nik et al., 2018). In our previous study, the BG vaccine was able to achieve over 90% immune protection against virulent challenges using the same serotype O2 strain, while it showed poor cross-protection against serotypes O1 and O78 (Hu et al., 2019). Further research is needed to provide cross-protection rates between serotypes.

## Conclusion and outlook

Avian pathogenic *E*. *coli* is considered responsible for multi-factorial illness and causes significant economic losses in the poultry industry over the world. APEC antibiotic resistance is serious, which increases the opportunity to transmit antibiotic resistance genes from APEC to human pathogens. Investigations are necessary to provide concrete evidence for the zoonotic transmission of APEC to humans. However, we highlight the crucial roles played by the different virulence factors; further investigations and studies are suggested to understand the contribution of virulence factors in APEC virulence. We should continue the efforts to identify more potential virulence factors and reveal the pathogenic mechanism, thus helping to develop a novel diagnosis method and vaccines to control avian colibacillosis. The virulence factors and drug resistance genes of APEC can be prevented from being transmitted to humans through food animals and endangering human health.

## Author contributions

JH, DA, and SW: conceptualization and original draft writing—review and editing. BZ, HZ, LY, WG, XW, ZW, DW, HP, MT, and JQ: helped in revising. All authors have read and agreed to the published version of the manuscript.
